# Integrating intrinsic musculoskeletal pathology and genetics: Recent advances in unravelling the causative factors of adolescent idiopathic scoliosis

**DOI:** 10.1016/j.bonr.2025.101882

**Published:** 2025-10-20

**Authors:** Ellie H. Northall, Liam M. Grover, Helen M. McGettrick, Matthew Newton Ede, Amy J. Naylor, Simon W. Jones

**Affiliations:** a Department of Inflammation and Ageing, MRC Versus Arthritis Centre for Musculoskeletal Ageing Research, University of Birmingham, Birmingham, B15 2TT, UK; b Healthcare Technology Institute, Department of Chemical Engineering, University of Birmingham, Birmingham, B15 2TT, UK; c The Royal Orthopaedic Hospital, Birmingham, B31 2AP, UK; d National Institute for Health and Care Research (NIHR) Birmingham Biomedical Research Centre, UK

**Keywords:** Scoliosis, Spine, GWAS, AIS, Musculoskeletal

## Abstract

Adolescent Idiopathic Scoliosis (AIS) is a common paediatric spinal disorder of incompletely understood aetiology. Current interventions include bracing and invasive surgery. However, determining which patients will benefit from observation, bracing, or surgery remains challenging due to the difficulty in predicting disease progression. A number of factors have previously been purported to play a causative role including hormones and biomechanics. However, recently GWAS and in vitro studies have provided insight into the underlying signalling pathways and intrinsic factors that act as drivers of AIS pathology across different tissue types, including spinal bone tissue, paraspinal muscles and cartilage. This review will explore these recent findings and evaluate their links to systemic factors as possible intrinsic drivers underpinning AIS pathophysiology and development.

## 1 Introduction

### 1.1 Prevalence and epidemiology

Idiopathic scoliosis is a complex three-dimensional structural deformity of the spine of unknown cause. Clinically defined (Weinstein et al., 2008) as a Cobb angle of greater than >10°, the associated lateral curvature and rotation of the vertebrae results in deformation, usually a “C” or “S” shape, of the spine. Idiopathic scoliosis can be further classified based on the age of presentation into infantile (0–3 years), juvenile (3–10 years) and adolescent (10–18 years) (Fletcher and Bruce, 2012; Choudhry et al., 2016). The latter, adolescent idiopathic scoliosis (AIS), is one of the most common spinal deformities in children, affecting 2–3 % of the general population (Tambe et al., 2018). This prevalence is subject to regional variation (Qiu, 2017), which could indicate the role of socioeconomic factors in pathogenesis, as recently discussed by Orellana et al. (Orellana et al., 2024). AIS has long been known to be influenced by sex, not only appearing to be more prevalent in females (Kamtsiuris et al., 2007; Daruwalla et al., 1985; Cilli et al., 2009; Nery et al., 2010) but also exhibiting more rapid and severe progression (Soucacos et al., 1997; Raggio, 2006; Luk et al., 2010; Ueno et al., 2011; Richards et al., 1994). Indeed, 10 times more female than male adolescents (Konieczny et al., 2013) present with Cobb angles >30°. There also appears to be a higher prevalence in individuals with lower body mass index (Zheng et al., 2017) and females with AIS have lower body weight and BMI than healthy counterparts (Ramírez et al., 2012; Tam et al., 2016), though studies incorporating more ethnic groups would support this further.

### 1.2 The clinical unmet needs of AIS patients

AIS patients do not typically present with any painful symptoms or neurological abnormalities and many children remain physically active despite the spinal deformation associated with the condition (Yagci and Yakut, 2019; Levi et al., 2019; van den Bogaart et al., 2019). The visible symptoms include a rib “hump” on the back, shoulder height asymmetry and torso “lean”, however in most cases these abnormalities do not place pressure on any organs or cause secondary symptoms but may lead to chronic pain and disability in adulthood (Ansari et al., 2024). Nevertheless, they may be distressing for the patient, with AIS patients reporting lower self-esteem and higher depression scores than age-matched general norms (Freidel et al., 2002).

The clinical management of AIS depends on both the severity of the curvature and whether the patient has previously received treatment (Lonstein, 2006). Current treatment strategies fall into three main categories and depend on both the risk of curve progression and the age of the patient. For moderate curvature (10–25°), patients are typically monitored at 3-month intervals via routine examination and X-ray radiography. Patients under observation are usually monitored until they reach skeletal maturity (16–18 years of age). Bracing is used for more significant curvature (25–40°) and has been shown to be highly effective in certain clinical trials, such as the Bracing in Adolescent Idiopathic Scoliosis Trial (BrAIST) (Weinstein et al., 2013), as long as compliance rates are high (Tang et al., 2024). However, compliance rates outside of clinical trials are variable with patients reporting that the devices are uncomfortable and interfere with their daily activities (Weinstein et al., 2013).

For skeletally immature patients with severe curvature (>40°), surgical intervention is recommended and typically seeks to both prevent further curve progression and attain some curve correction. Fusion surgical treatment uses metal implants attached to the spine and then attached to rods. Usually a posterior approach is taken, where the incision is made along the midline of the back. Less commonly, an anterior approach (where the incision is made through the front of the spine) (Al-Mohrej et al., 2020). Although studies have found no significant differences in morbidity between these surgical approaches (Carreon et al., 2007; Stepanovich et al., 2015), the associated morbidity rates are significant and range from 5.7 % (Coe et al., 2006) to as high as 15.4 % (Carreon et al., 2007), though more recent studies suggest the complication rates are falling (3.3 %) (Meyers et al., 2021). It is also of note that none of these currently available treatments address the underlying causative drivers of the condition.

## 2 Musculoskeletal pathology in scoliosis

**Fig. 1:**
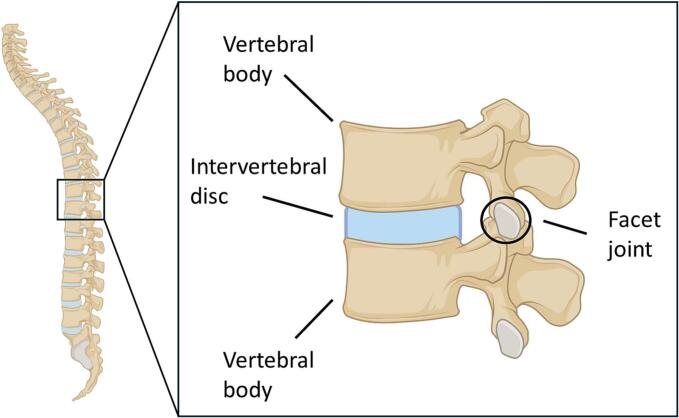
Anatomy of the human spine. Schematic of the individual vertebrae that make up the spine. Vertebral bodies are cushioned by intervertebral discs with facet joints providing additional support.

The spine functions to connect elements of the musculoskeletal system and, in turn, to aid movement. The spinal canal, consisting of 33 stacked vertebrae, is interspaced by intervertebral disks that cushion the vertebra and support tension absorption (Devereaux, 2007). The facet joints provide further flexibility and allow the vertebrae to slide against each other through cartilage cushioning. Soft tissues such as ligaments and muscles, connected to the bone via tendons, further support the spine and prevent buckling under load (Wagner et al., 2005). As such, mutations or erroneous function in any of these key musculoskeletal components may give rise to spinal deformities such as scoliosis (Fig. 1).

Approximately 60 % of female adolescent AIS patients have low bone mineral density (BMD) (Cheng et al., 2000; Hung et al., 2005). Low BMD is associated with more severe curves and a lower Risser grade (indicating less ossification), identifying osteopenia as an independent risk factor for curve progression in AIS (Sun et al., 2013). A study in female AIS patients further found that low BMD was associated with an abnormal bone quality profile indicated by (i) lower cortical bone area, (ii) cortical bone vBMD, (iii) trabecular number and (iv) greater trabecular separation, which collectively are indicative of endocortical remodelling, derangement in trabecular bone structure and disturbances in bone mineralization (Yu et al., 2014). More recently, these associations have been observed with abnormal bone microarchitecture suggestive of decreased endocortical bone apposition or active endocortical resorption, which could affect mechanical bone strength in AIS (Wang et al., 2017). It should be noted, however, that much of the current evidence linking AIS with low BMD comes from studies in Chinese cohorts. While similar trends have been reported in Japanese (Ishida et al., 2015; Nishida et al., 2023) and some American (Ramos et al., 2022) populations, the findings outside East Asia are less consistent. This raises the possibility that population-specific genetic haplotypes contribute to bone density differences in AIS, rather than this being a universal disease feature. Further multi-ethnic studies are needed to clarify whether reduced BMD is a global characteristic of AIS or reflects ethnicity-dependent susceptibility. In such individuals, calcium and Vitamin D supplementation have shown significant improvements in bone health. However, these positive effects dissipated after supplement was discontinued, illustrating the likely requirement for continuous dosing to sustain a therapeutic efficacy (Lam et al., 2021). Other studies have shown that supplementation may actually slow progression of AIS (Lam et al., 2017; Herdea et al., 2022).

## 3 Musculoskeletal pathology and cellular signalling pathways

As is evident from the condition's name, the underlying causes and mechanisms that drive idiopathic scoliosis are not fully established, but genetic factors have been identified– around 30 % of AIS patients have family members with the condition, with one study suggesting the association was closer to 97 % (Ogilvie et al., 2006) and the underlying genetic drivers have been recently collated in idiopathic scoliosis in general (Petrosyan et al., 2024), however the intrinsic factors underpinning AIS, in particular, are still not understood. Causative factors investigated include metabolic drivers (Acaroglu et al., 2012), hormonal imbalances, and also biomechanics, which have been extensively described in previous reviews (Hefti, 2013; Pizones et al., 2024).

Despite the genetic associations, the influence of intrinsic musculoskeletal factors has largely been overlooked as causative in AIS until recently. In this this review we have evaluated and collated recent advancements that shed light on the interplay between intrinsic mechanisms across key spinal tissues (including the paraspinal muscles, spinal bone, intervertebral disc, and cartilage) and the genetic background of AIS patients, with the aim of deepening understanding of the underlying causes driving the development of AIS.

### 3.1 General systemic genetics

Genome-Wide Association Studies (GWAS) have rapidly become a leading tool in recent years by enabling the linkage between genomic variants in patient cohorts with a particular disease or trait. Previously, GWAS has been used to identify locus *19p13* (Chan et al., 2002) and regions on chromosomes 6, 9, 16 and 17 (Miller et al., 2005) as susceptibility loci. More recently, whole exome sequencing has revealed 5q13.3 as a critical chromosome region with a single nucleotide variant in the *POC5* gene (p.A446T) (Patten et al., 2015) and another *POC5* variant (Xu et al., 2017) also associated with individuals with AIS.

### 3.2 Ciliary structure and function

*POC5* encodes the Proteome of Centriole 5 protein, essential for centriole assembly and elongation. An AIS-related mutation in the *POC5* gene has been shown to impair cell cycle, primary cilia length and centrosome protein interactions (Hassan et al., 2019). The genetic association of *POC5* with AIS derives from the findings that *POC5* mutants appear to have a higher prevalence in AIS patients compared to the general population (Mathieu et al., 2021a) and that the candidate region (9q31.2-q34.2) containing the *POC5* gene has also been identified by linkage analysis in AIS (Miller et al., 2012).

More recently, an insertion in the *TTLL11* gene within the 9q31.2-q34.2 locus has also been identified as the causative gene in one family (Mathieu et al., 2021b). *TTLL11* is a polyglutamylase from the tubulin tyrosine ligase-like (TTLL) family, which includes enzymes responsible for the post-translational modifications glutamylation and glycylation. Primary cilia, shown to mediate mechanosensing in bone (Malone et al., 2007), are defective in individuals with the mutation, reducing their number, size and the degree of polyglutamylation, compared to Wild type controls in experiments (Mathieu et al., 2021b). These findings position both POC5 and TTLL11 as candidates involved in initiating AIS pathology through disruption of cilia biology, rather than reflecting later curve changes.

### 3.3 Syndromic associations

Scoliosis is also highly prevalent in association with DiGeorge syndrome (Homans et al., 2019) (also known as 22q11.2 deletion syndrome), a common microdeletion syndrome that presents as immunodeficiency, hypoparathyroidism, and congenital heart disease. Around half of these patients will develop a scoliosis pathotype that highly resembles AIS. More recently, this has been proposed as a model of the development of early-onset scoliosis in humans (de Reuver et al., 2021) and a common pathological mechanism between DiGeorge and AIS is suggested to be the dysregulation of microRNA expression, specifically miR-93 and miR-1306 (Montemurro et al., 2022).

### 3.4 Neurotransmitter and inflammatory signalling

Another gene, tryptophan hydrolase 1 (*TPH1*), has long been investigated for its association with AIS (Wang et al., 2008), with differing conclusions (Nelson et al., 2011; Yablanski et al., 2016). A single nucleotide polymorphism (rs10488682) however, has recently been found to be statistically significant in correlation with AIS (Li et al., 2021). Lastly, a meta-analysis (Sobhan et al., 2020) has suggested there is a susceptibility link between an *IL-6* polymorphism (−174G>C) and the risk of developing AIS, after several previous studies gave inconsistent results (Nikolova et al., 2015; Sui et al., 2017; Lee et al., 2018). Both TPH1 and IL-6 variants may play a role in curve progression or severity, rather than initiation.

### 3.5 Paraspinal muscle-specific developments

Abnormal muscle growth and/or tropism has been implicated in AIS pathology due to the functional balance between muscles and bone that is essential for skeletal homeostasis. Indeed, biological maladaptation in the paraspinal muscle has previously been reported in AIS (Shahidi et al., 2021) and the bilateral paraxial muscles are important in the symmetrical growth and development of spinal curvature (Kikanloo et al., 2019). Recent studies suggest that subtle neuromuscular control defects may contribute to AIS initiation, indicating that AIS may not only be a consequence of mechanical imbalance but also of impaired neuromuscular signalling. Specifically, impaired glycine neurotransmission has been implicated as a potential neuropathic origin for idiopathic scoliosis, likely affecting central pattern generators (CPGs) responsible for spinal posture regulation (Wang et al., 2025, Wang et al., 2024).

#### 3.5.1 Causal vs consequence distinction

In AIS patients, significant imbalances in muscle volume and fatty depositions have been observed in the facets of the curve: on the convex side, larger muscle volume observed at the apex spinal level is consequential hypertrophy due to stretch-induced stress on satellite cells, whilst on the concave side, observed fatty deposition is understood to be fatty infiltration of muscle atrophy (Jiang et al., 2017). These changes are widely interpreted as secondary ‘results of the curve’ rather than primary causes.

The ligamentum flavum (LF) are a series of ligaments that connect the ventral parts of the laminae of adjacent vertebrae. Hypertrophy of these ligaments is seen in mechanical stress conditions (Fukuyama et al., 1995). Recently, LF hypertrophy has been observed in AIS patients, with the convex side significantly more hypertrophic than the concave side, and an increase in the expression of genes *ERC2* and *MAFB*, both of which were significantly associated with increased collagen expression on the convex side (Seki et al., 2022). Moreover, increased electrical activity of paraspinal muscle (PSM) on the convex side on the curve was observed (Stetkarova et al., 2016; Farahpour et al., 2015), with a higher numerical distribution of muscle fibre type 1 (Mannion et al., 1998; Gonyea et al., 1985). Recently, it has been shown that fibre type-specific pathological changes on the concave side of the spine are more severe compared to both the convex side and healthy controls (Shao et al., 2020), with the authors further showing that some of these characteristics (myonuclei density, cross sectional area and total and activated satellite cell density) are closely associated with curve severity. Furthermore, higher circulating levels of muscle structural proteins in progressive AIS possibly suggest structural damage at the microscopic level can be used as a biomarker for curve progression (Wang et al., 2021a). Again, these muscle changes are likely downstream consequences of spinal curvature, although certain susceptibility genes (see below) suggest muscles may also contribute to initiation.

#### 3.5.2 Genetic contributions to muscle development

Fibrillin-1 (*FBN1*) was identified by GWAS as a susceptible gene of AIS (Buchan et al., 2014) and more recently a common variant was significantly associated with AIS development in the Chinese population (Sheng et al., 2019). *FBN1* codes for a protein crucial to extracellular microfibril organization in skeletal muscle cells and a polymorphism may be considered a protective factor in AIS susceptibility (de Azevedo et al., 2022). The GWAS-identified functional variant rs35333564, located in the host gene of microRNA MIR4300 (MIR4300HG) is associated with AIS curve progression in a Japanese population (Ogura et al., 2017). This association has been replicated in Chinese patients, and it was speculated that *CRTC1* is the target gene of MIR4300 (Wang et al., 2021b). *CRTC1* is known to be expressed in skeletal muscle cells where it regulates bone metabolism (Kim et al., 2019). Another gene identified by GWAS, *LBX1*, has been implicated in the myogenesis of the paraspinal muscles in the etiopathology of AIS (Londono et al., 2014; Xu et al., 2021). *LBX1* is essential in regulating migration and proliferation of muscle precursor cells and is associated with AIS (Takahashi et al., 2011; Jiang et al., 2019). It has been suggested as one of the more promising candidate genes in AIS susceptibility, due to its possible functions in muscle progenitor cell migration and neuronal determination processes (Luo et al., 2021). Lastly, *Tent5a*, a noncanonical poly(A) polymerase associated in skeletal disorders, was previously identified as an AIS susceptibility gene (Kou et al., 2019). *Tent5a* was differentially expressed between the paravertebral muscles either side of the spine curve apex (convex and concave) and has further been shown to modulate muscle fibre formation in AIS via maintenance of myogenin expression (Luo et al., 2022). These genetic associations suggest that while most muscle tissue changes are secondary, specific variants in FBN1, LBX1, and Tent5a may predispose individuals to abnormal muscle development that contributes to AIS initiation.

### 3.6 Estrogen receptors in paraspinal muscles

Since AIS is both more prevalent and severe in females, it has long been postulated that hormones such as estrogen can influence its progression (Konieczny et al., 2013; Weinstein, 1999; Dayer et al., 2013). Estrogen acts on target cells through estrogen receptor 1 (ESR1) or 2 (ESR2) (Kuiper et al., 1996) and genetic mutations in both *ESR1* and *2* are associated with the risk of AIS development (Wang et al., 2020a). ESR2 is detected in skeletal muscle (Wiik et al., 2003) and paravertebral skeletal muscle tissue (Rusin et al., 2012), and its expression is thought to be dependent on methylation of its promoter (Zhao et al., 2003). Recently, the methylation level within the ESR2 promoter was found to be significantly greater in paraspinal muscles on the concave side of the curvature, compared to muscles on the convex side, showing an association with the occurrence (but not severity) of idiopathic scoliosis (Chmielewska et al., 2020). With ESR1, methylation of tissue dependent and differentially methylated regions (T-DMRs) was significantly higher in the superficial muscles compared to the deep paravertebral muscles (Janusz et al., 2021). Furthermore, ESR1 promoter methylation levels correlated with ESR1 expression on the concave, but not convex, side of the curvature (Janusz et al., 2021). ESR1 expression was significantly higher in deep paraspinal muscles compared to superficial ones and there is a left-right asymmetry of ESR1 and ESR2 expression in deep muscles (Kotwicki et al., 2022), with the authors further showing this asymmetry had a significant relationship with both the curve severity via Cobb angle and with the progression risk factor in AIS.

### 3.7 Disc and/or cartilage genetic developments

Previous genetic associations to cartilage biogenesis and intervertebral disk development in AIS have been extensively covered (Wise et al., 2020), however several more recent breakthroughs have provided further clarity. The *FAT3* gene, which co-ordinates cartilage differentiation and polarity (Le Pabic et al., 2014), has now been identified as a candidate AIS gene due to two rare protein-altering variants (Nada et al., 2022). Similarly, *PAX-1*, a transcription factor expressed in vertebral bodies and intervertebral discs – crucial for proper spinal structure formation, has associations with AIS (Sharma et al., 2015; Xu et al., 2018, Xu et al., 2019). Polymorphisms in *PAX-1* have now been associated with an increased risk of developing AIS and have been proposed as a potential biomarker (Pedrosa et al., 2022).

FLNB (filamin B) is a cytoplasmic protein active in chondrocytes that is involved in controlling and guiding proper skeletal development (Lu et al., 2007). Mutations in *FLNB* are associated with developmental disorders (Yang et al., 2016). A study using exome sequencing found multiple *FLNB* variants in AIS patients, with the authors suggesting AIS is an oligogenic disease (Jiang et al., 2020), where at least three loci are thought to influence a condition or disease trait. A study has also highlighted genetic pathways involving AIS-associated loci that regulate chondrogenesis, intervertebral disk (IVD) development and connective tissue maintenance and homeostasis (Makki et al., 2021). Lastly, female AIS patients have been shown to have lower androgen levels than age-matched controls, and further in vitro experiments suggested that this promoted abnormal cartilage development, possibly via the AR/IL-6/STAT3 signalling pathway (Wu et al., 2021).

### 3.8 Musculoskeletal metabolism developments

Evidence of intrinsic differences in spinal bone metabolism in AIS was reported by Pearson et al. (2019), who found that osteoblasts at the curve apex exhibited a differential metabolic capacity compared to osteoblasts from outside the curve (Pearson et al., 2019). Links between the relationship of bone metabolism and AIS have long been considered, with up to 72 metabolites differentially expressed in AIS patient plasma compared to healthy controls (Xiao et al., 2021). AIS patients have higher adiponectin levels and a lower leptin/adiponectin ratio than healthy controls and moderate cases, as well as higher resistin levels, suggesting a role for adipokines (Normand et al., 2022). Dipeptidyl peptidase-4 (DPP-4) modulates insulin-related metabolism (Matteucci and G., 2009) and has been shown to have lower serum expression in AIS patients (Normand et al., 2017). A more recent study found that aberrant DPP-4 expression impairs AIS myoblasts sensitivity to glucose and insulin and influences cell viability during myogenesis (Dai et al., 2022). miRNA expression profiles were analysed in severe and mild AIS cases and their osteoblasts extracted to determine their effects on bone metabolism – miR-151a-3p is overexpressed in severe cases and inhibits GREM1 (itself an inhibitor of BMPs in the TGF-β signalling pathway) so disrupts bone homeostasis (Wang et al., 2020b). The upregulation of another miRNA (miR-96-5p) has also recently been confirmed in AIS patients, with pathway analysis showing further functional links with bone metabolic pathways (Chen et al., 2022).

## 4 Discussion

**Fig. 2:**
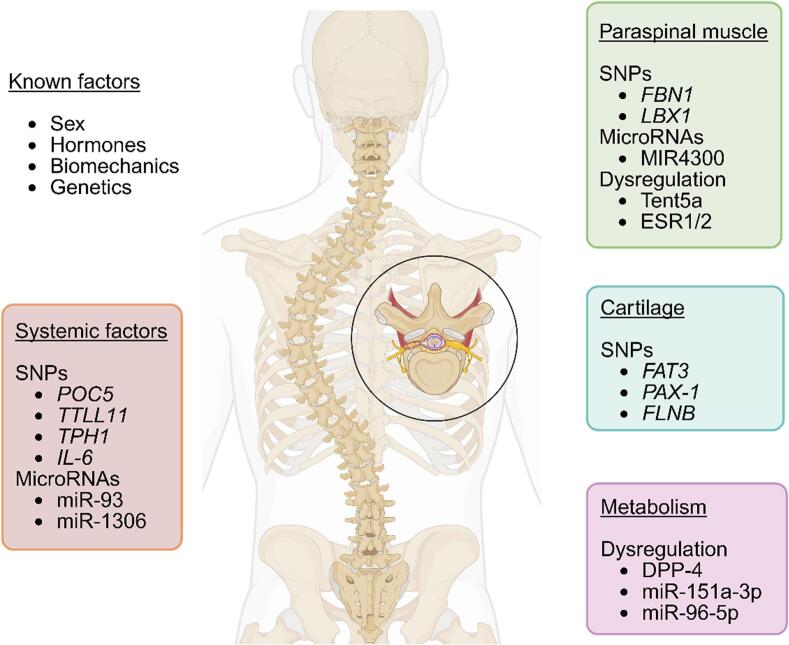
Recent genetic factors associated with AIS: Summary of the major genetic loci, genes, and signalling pathways that have been implicated in the multifactorial pathogenesis of Adolescent Idiopathic Scoliosis (AIS). Identified through genome-wide association studies (GWAS), candidate gene analyses, and functional studies, these genetic factors contribute to skeletal development, neuromuscular control, connective tissue integrity, and hormonal regulation.

AIS remains a multifactorial condition, driven by the complex interplay between musculoskeletal and genetic factors. Recent research has deepened our understanding of these mechanisms, but significant challenges remain in deciphering the full pathogenesis of AIS. This review has highlighted emerging evidence pointing toward intrinsic musculoskeletal pathologies—such as bone density deficits, muscle imbalances, and cartilage anomalies—as key contributors, alongside genetic predispositions, to AIS development (Fig. 2, Table 1).

**Table 1:** Key genetic variants associated with risk, progression or pathology of AIS.

Tissue	Gene	Pathology	Effect	Ref
Systemic	POC5	SNP	Impaired primary cilia and cell cycle	Hassan et al., 2019; Mathieu et al., 2021a
	TTLL11	SNP	Defective primary cilia	Mathieu et al., 2021b
	miR-93		Association with DiGeorge syndrome	Montemurro et al., 2022
	miR-1306			
	TPH1	SNP	Correlates with AIS severity	Li et al., 2021
	IL-6	SNP	Susceptibility link	Sobhan et al., 2020
Muscle	ERC2	Increased gene expression	Increased collagen expression at convex site	Seki et al., 2022
	MAFB			
	FBN1	GWAS	Associated with development	Sheng et al., 2019
	MIR4300	GWAS/SNP	Association with curve progression through targeting CRTC1	Ogura et al., 2017; Wang et al., 2021b
	LBX1	GWAS	Susceptibility link due to muscle cell migration	Luo et al., 2021
	Tent5a		Modulates muscle fibre formation in AIS	Luo et al., 2022
	ESR1	Asymmetry in expression	Associated with development	Wang et al., 2020a
	ESR2		Associated with curve severity	Kotwicki et al., 2022
Cartilage	FAT3	Protein altering variants	Candidate gene	Nada et al., 2022
	FLNB	Exome sequencing	Association through variants	Jiang et al., 2020
Disc	PAX-1	SNP	Associated with development risk/biomarker	Pedrosa et al., 2022
Serum	DPP-4	Lower expression in AIS	Impairs myoblasts sensitivity	Normand et al., 2017; Dai et al., 2022
Bone	miR-151a-3p	Overexpressed	Disrupts bone homeostasis through GREM1 inhibition	Wang et al., 2020b

Musculoskeletal abnormalities, particularly in bone quality, have emerged as critical to understanding AIS progression. Studies reveal that female AIS patients frequently exhibit low bone mineral density (BMD), with microarchitectural changes, such as reduced cortical bone area and increased trabecular separation, leading to structural weakening of the spine. These findings suggest that bone pathology, influenced by genetic factors, is integral to the mechanical dysfunction seen in AIS. The implications of bone density alterations, particularly in how they may predispose the spine to deformity under biomechanical stress, indicate a need to further explore how intrinsic skeletal characteristics contribute to curve progression in AIS.

Simultaneously, advances have uncovered the significance of muscle imbalances in AIS pathology. Paraspinal muscle asymmetry is a well-documented feature and the role of genes such as LBX1 and Tent5a, which are critical in muscle development and myogenesis, suggests that AIS is driven, at least in part, by muscle pathology that exacerbates spinal curvature. In this context, the discovery of MIR4300 (Ogura et al., 2017) and its role in regulating bone and muscle metabolism strengthens the hypothesis that these musculoskeletal imbalances are not merely secondary to spinal deformity but may be causative in nature.

Adding to this complexity is the influence of hormonal regulation, particularly estrogen, which has been linked to the gender disparity in AIS prevalence and severity. Genetic mutations affecting estrogen receptors ESR1 and ESR2, along with differential methylation patterns in paraspinal muscles, have been shown to correlate with disease occurrence. These findings suggest that hormonal dysregulation exacerbates biomechanical stress on the spine, possibly through modulating muscle and bone homeostasis. Given the striking gender differences in both AIS prevalence and progression, further exploration into the role of hormones, including estrogen, may reveal crucial links between genetic predisposition and the observed musculoskeletal abnormalities.

While these intrinsic factors - bone density, muscle imbalance - are increasingly well understood, the relationship between them and genetic drivers is less clear. Genome-wide association studies (GWAS) have identified several key genetic variants linked to AIS, such as POC5, TTLL11, and LBX1, which offer new insights into the cellular and molecular underpinnings of the disease. However, the exact interplay between these genetic factors and musculoskeletal abnormalities remains an area ripe for further research. Moreover, systemic factors, such as metabolic and hormonal imbalances, are emerging as important contributors to AIS. The dysregulation of adipokines and altered glucose metabolism in muscle cells provide a potential link between broader systemic conditions and local musculoskeletal changes in AIS patients. Such findings suggest that a holistic view of AIS pathogenesis, incorporating both local musculoskeletal and systemic metabolic factors, may be necessary to fully understand the disease.

This integrated approach could also open the door to novel therapeutic strategies. Genetic findings, such as the upregulation of the PRKG1 pathway (Hou et al., 2020) or the dysregulation of estrogen receptor activity, suggest potential molecular targets for intervention. Pharmacological treatments that address these underlying genetic and molecular defects could prove more effective in halting or even reversing the progression of AIS. Additionally, epigenetic therapies targeting either dysregulated metabolites, methylation patterns in estrogen receptors or other key genes could offer new ways to modulate disease progression.

## 5 Key findings, knowledge gaps, and future directions

In summary, three main findings emerge from our review of the literature on AIS: (1) intrinsic musculoskeletal abnormalities (low bone mineral density, muscle asymmetry, ligament hypertrophy) are consistently observed and likely interact with genetic predisposition; (2) several genes identified by GWAS and functional studies (POC5, TTLL11, LBX1, Tent5a, MIR4300) highlight potential causal pathways in skeletal and muscle biology; and (3) systemic metabolic and hormonal dysregulation (e.g., estrogen signalling, adipokines, glucose metabolism) may act as important modifiers of curve initiation and progression.

Despite these advances, there remain critical knowledge gaps: (a) limited causal evidence distinguishing primary drivers from secondary consequences of curve progression; (b) incomplete understanding of how genetic variants converge on shared molecular or biomechanical pathways; (c) lack of longitudinal, phenotype-rich studies to track curve initiation; and (d) underexplored integration of systemic metabolic states with local musculoskeletal pathology.

To address these gaps, future research should prioritize: (i) prospective longitudinal cohorts with standardized phenotyping to clarify temporality; (ii) mechanistic studies in physiologically relevant models (including for example multicellular human organoids and animal models) to test causality of candidate genes and pathways; (iii) multi-omic integration (genomics, metabolomics, epigenetics) to resolve complex interactions; and (iv) exploration of precision therapeutic strategies, including metabolic modulators, hormone-based therapies, and epigenetic interventions. By aligning basic mechanistic work with clinically relevant outcomes, these approaches could accelerate the translation of genetic and metabolic insights into targeted therapies.

In conclusion, while significant progress has been made in understanding the genetic and intrinsic factors driving AIS, further research is required to fully elucidate how these elements interact at both cellular and systemic levels. The identification of key genetic drivers and the growing body of evidence linking musculoskeletal abnormalities to disease progression provide promising avenues for future therapeutic development. By integrating genetic, biomechanical, and systemic factors into a cohesive model, we may better predict disease progression, identify novel biomarkers, and ultimately develop targeted treatments for this complex and multifaceted condition.

## CRediT authorship contribution statement

**Ellie H. Northall:** Writing – review & editing, Writing – original draft, Investigation, Formal analysis, Data curation. **Liam M. Grover:** Writing – review & editing, Supervision, Formal analysis. **Helen M. McGettrick:** Writing – review & editing, Supervision, Formal analysis. **Matthew Newton Ede:** Writing – review & editing, Investigation, Conceptualization. **Amy J. Naylor:** Writing – review & editing, Supervision, Formal analysis, Conceptualization. **Simon W. Jones:** Writing – review & editing, Supervision, Formal analysis, Conceptualization.

## Ethics

The manuscript is a review of existing literature and thus does not contain primary clinical studies or patient data.

## Funding

E.N PhD studentship was funded by the 10.13039/501100000855University of Birmingham.

## Declaration of competing interest

All authors declare no conflict of interest.

## Data Availability

No data was used for the research described in the article.
